# The Role of Host Genetic Signatures on Root–Microbe Interactions in the Rhizosphere and Endosphere

**DOI:** 10.3389/fpls.2018.01896

**Published:** 2018-12-19

**Authors:** Peng Yu, Frank Hochholdinger

**Affiliations:** Institute of Crop Science and Resource Conservation, Crop Functional Genomics, University of Bonn, Bonn, Germany

**Keywords:** endosphere, interaction, microbial diversity, rhizosphere, root

## Abstract

Microbiomes inhabiting plants are crucial for plant productivity and well-being. A plethora of interactions between roots, microbiomes, and soil shapes the self-organization of the microbial community associated with the root system. The rhizosphere (i.e., the soil close to the root surface) and endosphere (i.e., all inner root tissues) are critical interfaces for the exchange of resources between roots and the soil environment. In recent years, next-generation sequencing technologies have enabled systemic studies of root-associated microbiomes in the endosphere and interactions between roots and microbes at the root-soil interfaces. Genetic factors such as species and genotype of host plants are the driving force of microbial community differentiation and composition. In this mini-review, we will survey the role of these factors on plant–microbe interactions by highlighting the results of next-generation genomic and transcriptomic studies in the rhizosphere and endosphere of land plants. Moreover, environmental factors such as geography and soil type shape the microbiome. Relationships between the root-associated microbiome, architectural variations and functional switches within the root system determine the health and fitness of the whole plant system. A detailed understanding of plant–microbe interactions is of fundamental agricultural importance and significance for crop improvement by plant breeding.

## Introduction

In vascular plants, the root system is the main interface for interactions with the soil microbiome. Roots influence biological activity and diversity of microbes in the soil by secreting organic substances including amino acids, carbohydrates and organic acids as well as by depositing root cap border cells and polysaccharide mucilage to soil environments (Marschner, 1995). Thus, root systems provide nutrient-rich niches for microbes. The interactions of roots and microbes are also enhanced by the highly active uptake of water and soluble molecules by roots and by transporting them to the energy delivering green part of plants. Soil provides a vital habitat for microorganisms (Marschner, 1995). Genomic studies have indicated that the soil type has in general a stronger influence on the composition of the plant root microbiome in both the rhizosphere and the endosphere than the plant species (Bulgarelli et al., 2012; Peiffer et al., 2013; Schreiter et al., 2014). However, under identical soil conditions, the plant genotype drives the diversity of root microbial community structure and function, thus demonstrating that plants are able to filter their root microbiomes in a defined environment (Reinhold-Hurek and Hurek, 2011). Different plant species even genotypes within a species have shown distinct metabolic activities thus producing different types and amounts of organic compounds. Early studies have demonstrated that microbial communities of root systems varied between different plant genera (Smalla et al., 2001) and species (Tan et al., 2011). For instance, highly diverged angiosperm species vary with respect to bacterial diversity and composition in both the rhizosphere and endosphere (Fitzpatrick et al., 2018). In addition, the composition of root microbiomes can be divergent at the level of subspecies, as shown from different cultivars of potato (Sessitsch et al., 2002; Andreote et al., 2010) and rice (Hardoim et al., 2011). Comparative genomic analyses of microbial communities in the endosphere and rhizosphere, require suitable methods to efficiently separate these compartments along the complex morphological and anatomical structure of the root system (Reinhold-Hurek et al., 2015).

Recent studies have applied high-throughput DNA sequencing to study the composition and organization of different plant microbiomes (16S rRNA gene based community profiling), including both dicot (Bulgarelli et al., 2012; Lundberg et al., 2012; Schlaeppi et al., 2014) and monocot (Knief et al., 2012; Peiffer et al., 2013; Edwards et al., 2015; Walters et al., 2018) species. This mini review will highlight recent progress in deciphering the influence of the genetic background of host plants on the microbial diversity and composition in the rhizosphere and endosphere of the root system.

## Community Profiling of Host–Microbe Interaction in the Rhizosphere

The rhizosphere is the narrow soil zone directly surrounding the root system, which is highly modulated by the root system (Marschner, 1995). Roots release low-molecular-weight exudates, which decompose very quickly. Those exudates attract soil microorganisms into the rhizosphere where they multiply and enrich by several orders of magnitude compared to bulk soil (Sasse et al., 2017). Attracted microorganisms strongly influence plant nutrition by mineralizing organic nutrients and transformation of inorganic nutrients (Marschner, 1995). Moreover, host–microbe interaction studies have revealed that plants are able to recruit beneficial microbes to increase microbial activity and reduce pathogen attacks to maintain plant health in the rhizosphere (Berendsen et al., 2012). Recent surveys have revealed that different plant species can shape their rhizosphere microbiome, as shown from the host specific microbial communities grown under identical soil condition (Ofek-Lalzar et al., 2014). Rhizosphere microbiome composition has been suggested to be controlled by root exudate composition (Badri et al., 2009; Lakshmanan et al., 2012; Carvalhais et al., 2013; Lebeis et al., 2015). Particular plant species and even specific cultivars shape the composition of the rhizosphere microbiome (Peiffer et al., 2013; Turner et al., 2013; Ofek-Lalzar et al., 2014).

Human selection during domestication has significant reduced the genetic diversity of modern crop species in comparison to their progenitors (Doebley et al., 2006). Recent work has emphasized that domestication strongly shaped microbial diversity in the rhizosphere (Pérez-Jaramillo et al., 2016; Schmidt et al., 2016). Domesticated barley (*Hordeum vulgare*) shows a distinct microbiome compared to its wild progenitor with respect to function (Bulgarelli et al., 2015). Some of the genes affecting host–microbe interactions in domesticated barley show evidence of positive selection (Bulgarelli et al., 2015). Moreover, the maize progenitor teosinte shows significantly higher bacterial abundance and diversity in the rhizosphere compared to modern maize sweet corn and popcorn inbred lines (Szoboszlay et al., 2015). Nevertheless, a significant fraction of endophytic bacterial diversity observed in teosinte is conserved in modern hybrid maize (Johnston-Monje and Raizada, 2011; Johnston-Monje et al., 2014). In vegetable species as for instance beet and lettuce, rhizosphere bacterial community composition of wild ancestors is different from their corresponding modern varieties (Zachow et al., 2014; Cardinale et al., 2015). Similarly, genetically divergent wild and modern bean genotypes display distinct bacterial communities (Pérez-Jaramillo et al., 2017). Interestingly, recent findings indicate that fungal communities are stronger influenced by host genotypes than by bacterial communities in the rhizosphere during sunflower domestication and breeding (Leff et al., 2017). These results indicate that microbial composition and diversity in the rhizosphere co-evolved with host domestication and modern breeding but that it is also dependent on their own genetic and genomic identity in plants.

High-yielding maize hybrids display a significantly higher bacterial abundance and more elite probiotic rhizospheric strains with higher productivity of 2,4-diacetylphloroglucinol and superior root-colonization ability in the rhizosphere than their parental inbred lines (Picard and Bosco, 2006). It has been suggested that this is due to more secretion of exudates relative to their parental inbred lines (Picard et al., 2004, 2008; Picard and Bosco, 2005, 2006). Specifically, the antibiotic 2,4-diacetylphloroglucinol produced by probiotic rhizobacteria has been shown growth-promoting properties and inhibition of phytopathogenic bacteria and fungi in crops (Haas and Keel, 2003; Vacheron et al., 2013). In rice, enrichment of a particular group of *Alphaproteobacteria* and *Ascomycota* has been shown in hybrid cultivars in comparison to conventional cultivars (Hussain et al., 2011). These results suggest that hybrids display beneficial plant–microbe interactions that might be related to heterosis i.e., the superior vigor of these plants. Thus, it would be interesting to compare additional hybrids to survey how heterosis affects the root microbiome.

## Community Profiling of Host–Microbe Interaction in the Endosphere

The endosphere, which comprises all inner root tissues is inhabited by microbes (Reinhold-Hurek et al., 2015). Function and composition of microbial communities are very different between the rhizosphere and the endosphere. These observations indicate that the endosphere of plants has the potential to attract or filter the microbes inhabiting the rhizosphere (Edwards et al., 2015; Naylor et al., 2017). Microbial communities inhabiting the root endosphere also engage in symbiosis, which is defined as close and long-term biological interaction between two different biological organisms with their host. Endophytic communities of the root system are distinct assemblies and not mere subsets of the microbial communities in the rhizosphere (Gottel et al., 2011). Microbes have been successfully isolated from the endosphere by sonication in Arabidopsis (Bulgarelli et al., 2012; Lundberg et al., 2012). Recent experiments have demonstrated that the host genotypes have relatively little effects on the composition of bacteria in the endosphere of the Arabidopsis root (Bulgarelli et al., 2012; Lundberg et al., 2012). A study in rice has demonstrated that spatially separated parts of the endosphere harbor distinct and overlapping bacterial communities, which are affected by the genotype of the host plant (Edwards et al., 2015). These findings indicate that microbiomes are selected distinctively by the root interior and the rhizosphere in response to multiple signals from the plant. A systemic investigation of cereals suggests that bacterial communities of the host root endosphere correlate with host phylogenetic distance (Naylor et al., 2017). These findings highlight that the composition and diversity of microbial communities in the endosphere depend on specific species and genotypes under a certain geographical condition during the evolutionary history.

It has been shown that beneficial association between host and plant growth promoting rhizobacteria is an inherited trait in the endosphere of tomato plants (Smith et al., 1999). Moreover, it has been demonstrated that hybrids tend to enrich more phylogenetic subgroups of arbuscular mycorrhizal fungi in comparison to their parental inbred lines in the endosphere (Picard et al., 2008). In addition, arbuscular mycorrhizal colonization between inbred lines varies with diverse geographic origin and year of release in maize (An et al., 2010). Finally, modern maize hybrids have a substantially higher colonization rate with arbuscular mycorrhizal fungi than their parental inbred lines or landraces (An et al., 2010). These results substantiate the notion that modern breeding lead to beneficial association between maize and arbuscular mycorrhizal fungi in the endosphere.

In contrast to the model plant Arabidopsis and most dicot plant species, cereal plants display distinct root types including seminal roots and postembryonic shoot-borne crown and brace roots, which show diverse anatomical structures and transcriptomic signatures during development (Hochholdinger et al., 2018). Moreover, lateral roots contribute substantially to the overall surface for water and nutrient uptake in maize (Yu et al., 2016). Recent observations have demonstrated that distinct functional and metabolic characteristics of different root types significantly influence root-inhabiting microbiomes in both maize and Brachypodium (Kawasaki et al., 2016; Yu et al., 2018). These results highlight, that microbiomes diverge even between distinct regions of the root system (Yu et al., 2018). Moreover, some earlier studies have shown that metabolic and genetic fingerprinting along the root are highly diversified for different plant species (Yang and Crowley, 2000; Baudoin et al., 2002). This results in distinct microbial signatures along the different root zones (Kawasaki et al., 2016). These discoveries demonstrate that distinct root microbiomes of specific root types will be averaged out by studying the microbial community structure in a whole root system (Kawasaki et al., 2016). Therefore, it will be necessary to compare root type-specific microbial communities between dicot and monocot species. This will provide an evolutionary perspective to the understanding of how developmental characteristics affect the microbiome in the endosphere and rhizosphere.

## Conclusion and Perspectives

Microbial diversity of the rhizosphere and endosphere is regulated by physical and chemical characteristics of the rhizosphere. These are partly determined by the species and genotype of the host root. Nevertheless, it is still unclear whether the phylogenetic distance between genotypes is correlated with the microbial community composition in the rhizosphere (Bouffaud et al., 2012, 2014; Peiffer et al., 2013; Schlaeppi et al., 2014). Differences in microbial community composition might only be observed between genotypes which display large genetic distances. For example, domesticated genotypes usually display distinct microbiome compositions in comparison with their wild progenitor (Schmidt et al., 2016). Similarly, genetically diverse modern inbred lines also display largely distinct microbiomes (Peiffer et al., 2013; Walters et al., 2018). Another instrumental factor determining the divergence of microbiome composition between different genotypes within a species is the sampling strategy to harvest the rhizosphere and endosphere. Many rhizosphere sampling strategies are likely not reproducible enough because of their low resolution. Recent studies in maize and Brachypodium indicate, that different root types harbor different microbial communities (Kawasaki et al., 2016; Yu et al., 2018), indicating microbiome variations even in the same organ of a single plant. Longitudinal separation of developmentally diverse root zones (root tip, elongation zone, differentiation zone and maturation zone) in single root types will be beneficial to comprehensively detect the development-dependent microbial gradients of host–microbe interaction. In addition, fine-scale isolation of rhizosphere and endosphere by laser capture microdissection in combination with next-generation sequencing would be an important tool to systemically target the direct interaction between plants and microbes at the cellular level.

## Author Contributions

Both PY and FH contributed to the writing of this mini review.

## Conflict of Interest Statement

The authors declare that the research was conducted in the absence of any commercial or financial relationships that could be construed as a potential conflict of interest.

## References

[B1] An G. H. Kobayashi S. Enoki H. Sonobe K. Muraki M. Karasawa T. (2010). How does arbuscular mycorrhizal colonization vary with host plant genotype? An example based on maize (*Zea mays*) germplasms. *Plant Soil* 327 441–453. 10.1007/s11104-009-0073-3

[B2] Andreote F. D. Rocha U. N. Araujo W. L. Azevedo J. L. van Overbeek L. S. (2010). Effect of bacterial inoculation, plant genotype and developmental stage on root-associated and endophytic bacterial communities in potato (*Solanum tuberosum*). *Antonie Van Leeuwenhoek* 97 389–399. 10.1007/s10482-010-9421-9 20352404 PMC2847171

[B3] Badri D. V. Quintana N. El Kassis E. G. Kim H. K. Choi Y. H. Sugiyama A. (2009). An ABC transporter mutation alters root exudation of phytochemicals that provoke an overhaul of natural soil microbiota. *Plant Physiol.* 151 2006–2017. 10.1104/pp.109.147462 19854857 PMC2785968

[B4] Baudoin E. Benizri E. Guckert A. (2002). Impact of growth stage on the bacterial community structure along maize roots, as determined by metabolic and genetic fingerprinting. *Appl. Soil Ecol.* 19 135–145. 10.1016/S0929-1393(01)00185-8

[B5] Berendsen R. L. Pieterse C. M. Bakker P. A. (2012). The rhizosphere microbiome and plant health. *Trends Plant Sci.* 17 478–486. 10.1016/j.tplants.2012.04.001 22564542

[B6] Bouffaud M. L. Kyselková M. Gouesnard B. Grundmann G. Muller D. Moënne-Loccoz Y. V. A. N. (2012). Is diversification history of maize influencing selection of soil bacteria by roots? *Mol. Ecol.* 21 195–206. 10.1111/j.1365-294X.2011.05359.x 22126532

[B7] Bouffaud M. L. Poirier M. A. Muller D. Moënne-Loccoz Y. (2014). Root microbiome relates to plant host evolution in maize and other Poaceae. *Environ. Microbiol.* 16 2804–2814. 10.1111/1462-2920.12442 24588973

[B8] Bulgarelli D. Garrido-Oter R. Münch P. C. Weiman A. Dröge J. Pan Y. (2015). Structure and function of the bacterial root microbiota in wild and domesticated barley. *Cell Host Microbe* 17 392–403. 10.1016/j.chom.2015.01.011 25732064 PMC4362959

[B9] Bulgarelli D. Rott M. Schlaeppi K. Ver Loren van Themaat E. Ahmadinejad N. (2012). Revealing structure and assembly cues for Arabidopsis root-inhabiting bacterial microbiota. *Nature* 488 91–95. 10.1038/nature11336 22859207

[B10] Cardinale M. Grube M. Erlacher A. Quehenberger J. Berg G. (2015). Bacterial networks and co-occurrence relationships in the lettuce root microbiota. *Environ. Microbiol.* 17 239–252. 10.1111/1462-2920.12686 25367329

[B11] Carvalhais L. C. Dennis P. G. Fan B. Fedoseyenko D. Kierul K. Becker A. (2013). Linking plant nutritional status to plant-microbe interactions. *PLoS One* 8:e68555. 10.1371/journal.pone.0068555 23874669 PMC3713015

[B12] Doebley J. F. Gaut B. S. Smith B. D. (2006). The molecular genetics of crop domestication. *Cell* 127 1309–1321. 10.1016/j.cell.2006.12.006 17190597

[B13] Edwards J. Johnson C. Santos-Medellín C. Lurie E. Podishetty N. K. Bhatnagar S. (2015). Structure, variation, and assembly of the root-associated microbiomes of rice. *Proc. Natl. Acad. Sci. U.S.A.* 112 911–920. 10.1073/pnas.1414592112 25605935 PMC4345613

[B14] Fitzpatrick C. R. Copeland J. Wang P. W. Guttman D. S. Kotanen P. M. Johnson M. T. (2018). Assembly and ecological function of the root microbiome across angiosperm plant species. *Proc. Natl. Acad. Sci. U.S.A.* 115 1157–1165. 10.1073/pnas.1717617115 29358405 PMC5819437

[B15] Gottel N. R. Castro H. F. Kerley M. Yang Z. Pelletier D. A. Podar M. (2011). Distinct microbial communities within the endosphere and rhizosphere of *Populus deltoides* roots across contrasting soil types. *Appl. Environ. Microbiol.* 77 5934–5944. 10.1128/AEM.05255-11 21764952 PMC3165402

[B16] Haas D. Keel C. (2003). Regulation of antibiotic production in root-colonizing Pseudomonas spp. and relevance for biological control of plant disease. *Annu. Rev. Phytopathol.* 41 117–153. 10.1146/annurev.phyto.41.052002.095656 12730389

[B17] Hardoim P. R. Andreote F. D. Reinhold-Hurek B. Sessitsch A. van Overbeek L. S. van Elsas J. D. (2011). Rice root-associated bacteria: insights into community structures across 10 cultivars. *FEMS Microbiol. Ecol.* 77 154–164. 10.1111/j.1574-6941.2011.01092.x 21426364 PMC4339037

[B18] Hochholdinger F. Yu P. Marcon C. (2018). Genetic control of root system development in maize. *Trends Plant Sci.* 23 79–88. 10.1016/j.tplants.2017.10.004 29170008

[B19] Hussain Q. Liu Y. Zhang A. Pan G. Li L. Zhang X. (2011). Variation of bacterial and fungal community structures in the rhizosphere of hybrid and standard rice cultivars and linkage to CO2 flux. *FEMS Microbiol. Ecol.* 78 116–128. 10.1111/j.1574-6941.2011.01128.x 21569061

[B20] Johnston-Monje D. Mousa W. K. Lazarovits G. Raizada M. N. (2014). Impact of swapping soils on the endophytic bacterial communities of pre-domesticated, ancient and modern maize. *BMC Plant Biol.* 14:233. 10.1186/s12870-014-0233-3 25227492 PMC4189167

[B21] Johnston-Monje D. Raizada M. N. (2011). Conservation and diversity of seed associated endophytes in Zea across boundaries of evolution, ethnography and ecology. *PLoS One* 6:e20396. 10.1371/journal.pone.0020396 21673982 PMC3108599

[B22] Kawasaki A. Donn S. Ryan P. R. Mathesius U. Devilla R. Jones A. (2016). Microbiome and exudates of the root and rhizosphere of Brachypodium distachyon, a model for wheat. *PLoS ONE* 11:e0164533. 10.1371/journal.pone.0164533 27727301 PMC5058512

[B23] Knief C. Delmotte N. Chaffron S. Stark M. Innerebner G. Wassmann R. (2012). Metaproteogenomic analysis of microbial communities in the phyllosphere and rhizosphere of rice. *ISME J.* 6 1378–1390. 10.1038/ismej.2011.192 22189496 PMC3379629

[B24] Lakshmanan V. Kitto S. L. Caplan J. L. Hsueh Y. H. Kearns D. B. Wu Y. S. (2012). Microbe-associated molecular patterns-triggered root responses mediate beneficial rhizobacterial recruitment in Arabidopsis. *Plant Physiol.* 160 1642–1661. 10.1104/pp.112.200386 22972705 PMC3486800

[B25] Lebeis S. L. Paredes S. H. Lundberg D. S. Breakfield N. Gehring J. McDonald M. (2015). Salicylic acid modulates colonization of the root microbiome by specific bacterial taxa. *Science* 349 860–864. 10.1126/science.aaa8764 26184915

[B26] Leff J. W. Lynch R. C. Kane N. C. Fierer N. (2017). Plant domestication and the assembly of bacterial and fungal communities associated with strains of the common sunflower, *Helianthus annuus*. *New Phytol.* 214 412–423. 10.1111/nph.14323 27879004

[B27] Lundberg D. S. Lebeis S. L. Paredes S. H. Yourstone S. Gehring J. Malfatti S. (2012). Defining the core *Arabidopsis thaliana* root microbiome. *Nature* 488 86–90. 10.1038/nature11237 22859206 PMC4074413

[B28] Marschner H. (1995). *Mineral Nutrition of Higher Plants.* London: Academic Press.

[B29] Naylor D. DeGraaf S. Purdom E. Coleman-Derr D. (2017). Drought and host selection influence bacterial community dynamics in the grass root microbiome. *ISME J.* 11 2691–2704. 10.1038/ismej.2017.118 28753209 PMC5702725

[B30] Ofek-Lalzar M. Sela N. Goldman-Voronov M. Green S. J. Hadar Y. Minz D. (2014). Niche and host-associated functional signatures of the root surface microbiome. *Nat. Commun.* 5:4950. 10.1038/ncomms5950 25232638

[B31] Peiffer J. A. Spor A. Koren O. Jin Z. Tringe S. G. Dangl J. L. (2013). Diversity and heritability of the maize rhizosphere microbiome under field conditions. *Proc. Natl. Acad. Sci. U.S.A.* 110 6548–6553. 10.1073/pnas.1302837110 23576752 PMC3631645

[B32] Pérez-Jaramillo J. E. Carrión V. J. Bosse M. Ferrão L. F. de Hollander M. Garcia A. A. (2017). Linking rhizosphere microbiome composition of wild and domesticated *Phaseolus vulgaris* to genotypic and root phenotypic traits. *ISME J.* 11 2244–2257. 10.1038/ismej.2017.85 28585939 PMC5607367

[B33] Pérez-Jaramillo J. E. Mendes R. Raaijmakers J. M. (2016). Impact of plant domestication on rhizosphere microbiome assembly and functions. *Plant Mol. Biol.* 90 635–644. 10.1007/s11103-015-0337-7 26085172 PMC4819786

[B34] Picard C. Baruffa E. Bosco M. (2008). Enrichment and diversity of plant-probiotic microorganisms in the rhizosphere of hybrid maize during four growth cycles. *Soil Biol. Biochem.* 40 106–115. 10.1016/j.soilbio.2007.07.011

[B35] Picard C. Bosco M. (2005). Maize heterosis affects the structure and dynamics of indigenous rhizospheric auxins-producing *Pseudomonas* populations. *FEMS Microbiol. Ecol.* 53 349–357. 10.1016/j.femsec.2005.01.007 16329954

[B36] Picard C. Bosco M. (2006). Heterozygosis drives maize hybrids to select elite 2, 4-diacethylphloroglucinol-producing *Pseudomonas* strains among resident soil populations. *FEMS Microbiol. Ecol.* 58 193–204. 10.1111/j.1574-6941.2006.00151.x 17064261

[B37] Picard C. Frascaroli E. Bosco M. (2004). Frequency and biodiversity of 2,4-diacetylphloroglucinol-producing rhizobacteria are differentially affected by the genotype of two maize inbred lines and their hybrid. *FEMS Microbiol. Ecol.* 49 207–215. 10.1016/j.femsec.2004.03.016 19712415

[B38] Reinhold-Hurek B. Bünger W. Burbano C. S. Sabale M. Hurek T. (2015). Roots shaping their microbiome: global hotspots for microbial activity. *Ann. Rev. Phytopathol.* 53 403–424. 10.1146/annurev-phyto-082712-102342 26243728

[B39] Reinhold-Hurek B. Hurek T. (2011). Living inside plants: bacterial endophytes. *Curr. Opin. Plant Biol.* 14 435–443. 10.1016/j.pbi.2011.04.004 21536480

[B40] Sasse J. Martinoia E. Northen T. (2017). Feed your friends: do plant exudates shape the root microbiome? *Trends Plant Sci.* 23 25–41. 10.1016/j.tplants.2017.09.003 29050989

[B41] Schlaeppi K. Dombrowski N. Oter R. G. van Themaat E. V. L. Schulze-Lefert P. (2014). Quantitative divergence of the bacterial root microbiota in Arabidopsis thaliana relatives. *Proc. Natl. Acad. Sci. U.S.A.* 111 585–592. 10.1073/pnas.1321597111 24379374 PMC3896156

[B42] Schmidt J. E. Bowles T. M. Gaudin A. (2016). Using ancient traits to convert soil health into crop yield: impact of selection on maize root and rhizosphere function. *Front. Plant Sci.* 7:373. 10.3389/fpls.2016.00373 27066028 PMC4811947

[B43] Schreiter S. Ding G. C. Heuer H. Neumann G. Sandmann M. Grosch R. (2014). Effect of the soil type on the microbiome in the rhizosphere of field-grown lettuce. *Front. Microbiol.* 5:144 10.3389/fmicb.2014.00144 PMC3986527 24782839

[B44] Sessitsch A. Reiter B. Pfeifer U. Wilhelm E. (2002). Cultivation-independent population analysis of bacterial endophytes in three potato varieties based on eubacterial and Actinomycetes-specific PCR of 16S rRNA genes. *FEMS Microbiol. Ecol.* 39 23–32. 10.1111/j.1574-6941.2002.tb00903.x 19709181

[B45] Smalla K. Wieland G. Buchner A. Zock A. Parzy J. Kaiser S. (2001). Bulk and rhizosphere soil bacterial communities studied by denaturing gradient gel electrophoresis: plant-dependent enrichment and seasonal shifts revealed. *Appl. Environ. Microbiol.* 67 4742–4751. 10.1128/AEM.67.10.4742-4751.2001 11571180 PMC93227

[B46] Smith K. P. Handelsman J. Goodman R. M. (1999). Genetic basis in plants for interactions with disease-suppressive bacteria. *Proc. Natl. Acad. Sci. U.S.A.* 96 4786–4790. 10.1073/pnas.96.9.4786 10220371 PMC21769

[B47] Szoboszlay M. Lambers J. Chappell J. Kupper J. V. Moe L. A. McNear D. H. Jr. (2015). Comparison of root system architecture and rhizosphere microbial communities of balsas teosinte and domesticated corn cultivars. *Soil Biol. Biochem.* 80 34–44. 10.1016/j.soilbio.2014.09.001

[B48] Tan F. X. Wang J. W. Chen Z. N. Feng Y. J. Chi G. L. Rehman S. U. (2011). Assessment of the arbuscular mycorrhizal fungal community in roots and rhizosphere soils of Bt corn and their non-Bt isolines. *Soil Biol. Biochem.* 43 2473–2479. 10.1016/j.soilbio.2011.08.014

[B49] Turner T. R. Ramakrishnan K. Walshaw J. Heavens D. Alston M. Swarbreck D. (2013). Comparative metatranscriptomics reveals kingdom level changes in the rhizosphere microbiome of plants. *ISME J.* 7 2248–2258. 10.1038/ismej.2013.119 23864127 PMC3834852

[B50] Vacheron J. Desbrosses G. Bouffaud M. L. Touraine B. Moënne-Loccoz Y. Muller D. (2013). Plant growth-promoting rhizobacteria and root system functioning. *Front. Plant Sci.* 4:356. 10.3389/fpls.2013.00356 24062756 PMC3775148

[B51] Walters W. A. Jin Z. Youngblut N. Wallace J. G. Sutter J. Zhang W. (2018). Large-scale replicated field study of maize rhizosphere identifies heritable microbes. *Proc. Natl. Acad. Sci. U.S.A.* 115 7368–7373. 10.1073/pnas.1800918115 29941552 PMC6048482

[B52] Yang C. H. Crowley D. E. (2000). Rhizosphere microbial community structure in relation to root location and plant iron nutritional status. *Appl. Environ. Microbiol.* 66 345–351. 10.1128/AEM.66.1.345-351.2000 10618246 PMC91828

[B53] Yu P. Gutjahr C. Li C. Hochholdinger F. (2016). Genetic control of lateral root formation in cereals. *Trends Plant Sci.* 21 951–961. 10.1016/j.tplants.2016.07.011 27524642

[B54] Yu P. Wang C. Baldauf J. A. Tai H. Gutjahr C. Hochholdinger F. (2018). Root type and soil phosphate determine the taxonomic landscape of colonizing fungi and the transcriptome of field-grown maize roots. *New Phytol.* 217 1240–1253. 10.1111/nph.14893 29154441

[B55] Zachow C. Müller H. Tilcher R. Berg G. (2014). Differences between the rhizosphere microbiome of *Beta vulgaris* ssp. *maritima*-ancestor of all beet crops-and modern sugar beets. *Front. Microbiol.* 5:415. 10.3389/fmicb.2014.00415 25206350 PMC4144093

